# Bandgap Engineering of Indium Phosphide-Based Core/Shell Heterostructures Through Shell Composition and Thickness

**DOI:** 10.3389/fchem.2018.00567

**Published:** 2018-11-20

**Authors:** Reyhaneh Toufanian, Andrei Piryatinski, Andrew H. Mahler, Radhika Iyer, Jennifer A. Hollingsworth, Allison M. Dennis

**Affiliations:** ^1^Division of Materials Science and Engineering, Boston University, Boston, MA, United States; ^2^Theoretical Division, Los Alamos National Laboratory, Los Alamos, NM, United States; ^3^Department of Biomedical Engineering, Boston University, Boston, MA, United States; ^4^Los Alamos National Laboratory, Materials Physics and Applications Division, Center for Integrated Nanotechnologies, Los Alamos, NM, United States

**Keywords:** quantum dots, bandgap tunability, optoelectronic properties, photoluminescence, type-I quantum dot, type-II quantum dot, successive ion layer adsorption and reaction (SILAR)

## Abstract

The large bulk bandgap (1.35 eV) and Bohr radius (~10 nm) of InP semiconductor nanocrystals provides bandgap tunability over a wide spectral range, providing superior color tuning compared to that of CdSe quantum dots. In this paper, the dependence of the bandgap, photoluminescence emission, and exciton radiative lifetime of core/shell quantum dot heterostructures has been investigated using colloidal InP core nanocrystals with multiple diameters (1.5, 2.5, and 3.7 nm). The shell thickness and composition dependence of the bandgap for type-I and type-II heterostructures was observed by coating the InP core with ZnS, ZnSe, CdS, or CdSe through one to ten iterations of a successive ion layer adsorption and reaction (SILAR)-based shell deposition. The empirical results are compared to bandgap energy predictions made with effective mass modeling. Photoluminescence emission colors have been successfully tuned throughout the visible and into the near infrared (NIR) wavelength ranges for type-I and type-II heterostructures, respectively. Based on sizing data from transmission electron microscopy (TEM), it is observed that at the same particle diameter, average radiative lifetimes can differ as much as 20-fold across different shell compositions due to the relative positions of valence and conduction bands. In this direct comparison of InP/ZnS, InP/ZnSe, InP/CdS, and InP/CdSe core/shell heterostructures, we clearly delineate the impact of core size, shell composition, and shell thickness on the resulting optical properties. Specifically, Zn-based shells yield type-I structures that are color tuned through core size, while the Cd-based shells yield type-II particles that emit in the NIR regardless of the starting core size if several layers of CdS(e) have been successfully deposited. Particles with thicker CdS(e) shells exhibit longer photoluminescence lifetimes, while little shell-thickness dependence is observed for the Zn-based shells. Taken together, these InP-based heterostructures demonstrate the extent to which we are able to precisely tailor the material properties of core/shell particles using core/shell dimensions and composition as variables.

## Introduction

Semiconductor quantum dots (QDs) are used to study the fundamental photophysics of quantum confined systems, as well as, for numerous applications in photovoltaics, solid state lighting, biomedical imaging, and biosensing (Dennis et al., 2012b; Chuang et al., 2014; Lan et al., 2014; Ten Cate et al., 2015; Kagan et al., 2016; Hong et al., 2017; Chandran et al., 2018; Kong L. et al., 2018; McHugh et al., 2018). Quantum confinement is easily observed in simple, single semiconductor nanocrystals (NCs): smaller cores are more quantum confined, resulting in an increase in the energy gap between the conduction and valence bands as observed through higher energy (bluer) photon emission following photoexcitation. Core/shell heterostructures exhibit more complex size and compositional dependencies as the bandgap of the particle depends on both the position and relative offsets of the conduction and valence bands of the separate components (Murray et al., 1993; Alivisatos, 1996; Peng and Peng, 2001; Reiss et al., 2009). This added complexity enables significant tuning of the optoelectronic properties of the NCs well beyond what would be feasible with the individual components in isolation. Thus, the core/shell heterostructure can be designed to exhibit photoluminescent (PL) energies and lifetimes that are not achievable with discrete NCs of either the core or shell material alone. We explore the extent of this heterostructure tunability using indium phosphide cores and four different shell materials, zinc sulfide, zinc selenide, cadmium sulfide, and cadmium selenide.

Indium phosphide QDs have garnered significant interest as a cadmium-free option that is tunable throughout the visible wavelength regime (Micic et al., 1994; Guzelian et al., 1996; Battaglia and Peng, 2002; Xie et al., 2007; Li and Reiss, 2008; Xu et al., 2008; Tamang et al., 2016). As-synthesized InP QDs, however, are non-emissive due to non-radiative relaxation of excitation generated charge carriers facilitated by trapping at surface defects (Dennis et al., 2012a; Omogo et al., 2013; Balan et al., 2017; Reid et al., 2018). Fabrication of emissive cores can be performed through two main synthetic routes: etching the surface of the nanoparticle using a fluoride, or overcoating with a larger bandgap semiconductor to create a core/shell heterostructure (Danek et al., 1996; Dabbousi et al., 1997; Talapin et al., 2002; Adam et al., 2005; Chen et al., 2013; Mordvinova et al., 2014, 2017). The latter approach renders QDs emissive through passivation of surface trap states at the core/shell interface, thus inhibiting non-radiative recombination pathways while protecting the core nanocrystal against photo-oxidation (Battaglia et al., 2003). The bandgap of the shell material, as well as the position of its conduction and valence bands relative to those of the core NC, dictates the bandgap structure of the resulting heterostructure and, thereby, the resulting optoelectronic properties (Li and Wang, 2004; Wang et al., 2018).

**Figure 1:**
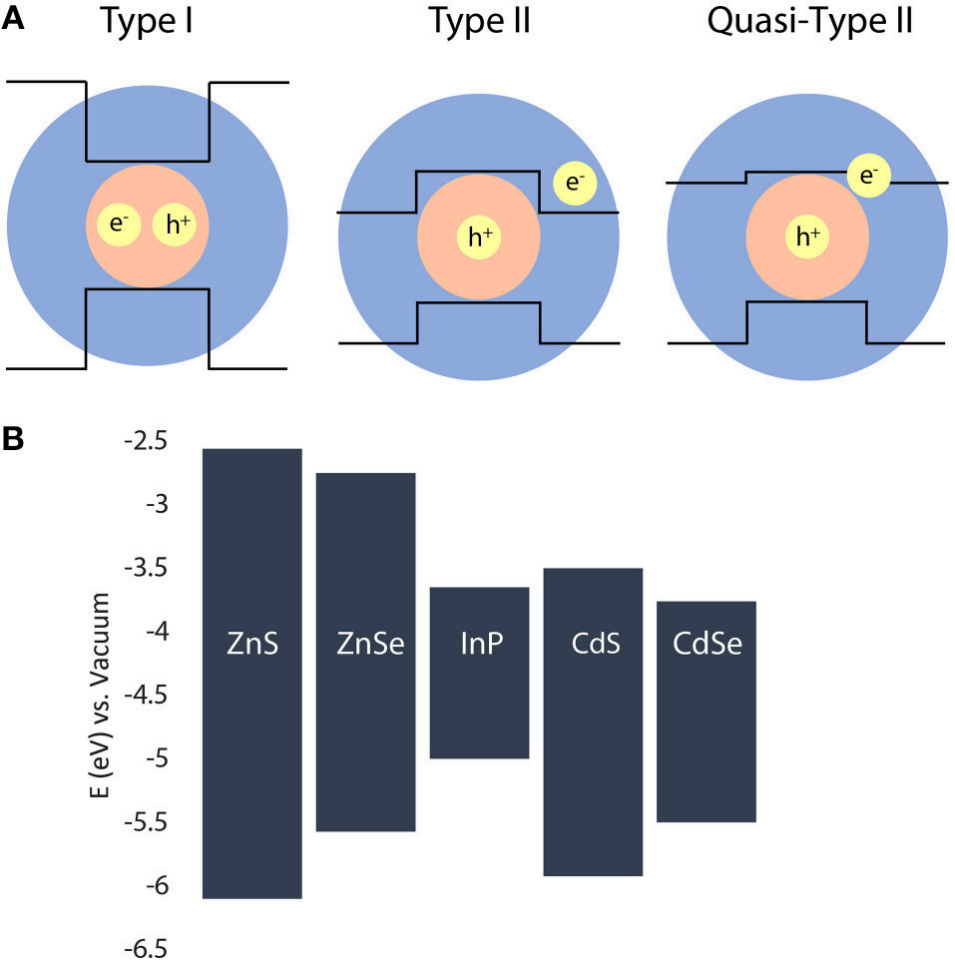
**(A)** Schematic representation of the relative positions of conduction and valence bands in different core/shell heterostructures. **(B)** Relative positions of the conduction and valance bands of the semiconductors used in this study (Wei and Zunger, 1998; Reiss et al., 2009).

In traditional type-I QDs, a low bandgap core material is coated with a second semiconductor with a wider bandgap than the core material with higher energy valence and conduction band offsets (Chandrasekaran et al., 2017; Brodu et al., 2018). Sufficiently large core size allows both the electron and hole to reside within the core, constituting the so-called type-I localization regime. A decrease in the core radius can eventually allow one charge carrier to delocalize into the shell while the other stays localized, especially if the band offset for either the conduction or the valence bands is small. This situation is distinguished as the quasi-type-II localization regime (Kaledin et al., 2018; Kong D. et al., 2018). In the prototypical example of CdSe/CdS, the core/shell conduction band offset is small, such that the core/shell structure exhibits a quasi-type-II localization regime with the electron wavefunction spreading from the core into the shell material, while the wavefunction of the heavy hole is still confined to the core (Kaledin et al., 2018; Kong D. et al., 2018). In cases where either the conduction or valance band of the shell lies within the bandgap of the core, a type-II localization regime is observed, whereby the electron and hole carriers are spatially separated into the core and shell (Dennis et al., 2012a; Acharya et al., 2015). As each carrier relaxes to its lowest energy state prior to radiative recombination, the resulting bandgap of the heterostructure is smaller than the bulk bandgap of either the core or shell material (Figure 1). Generally, the size and composition of a core/shell heterostructure dictates different localization regimes and subsequently determines photophysical properties such as the emission energy/wavelength/color and the photoluminescent (PL) lifetime (Reid et al., 2018).

InP QDs are most frequently shelled with ZnS to provide a protective, passivating layer yielding PL (Tessier et al., 2015). The high lattice mismatch between InP and ZnS (7.8%), however, can result in the introduction of interfacial stresses at the core/shell barrier, leading to the formation of defects and non-radiative recombination pathways. A shell of ZnSe offers a significantly reduced lattice mismatch and still induces PL, but is itself prone to photo-oxidation, making the final material prone to degradation over time. The current preferred solution is to use a multilayered or alloyed shell of ZnSe then ZnS to satisfy the requirements of low lattice mismatch at the core/shell interface and high stability at the shell/media interface (Ippen et al., 2012; Lim et al., 2013). Shelling with other semiconductor materials changes the band alignment and more dramatically impacts the particle photophysics (Htoon et al., 2010; Vela et al., 2010). For example, we produced type-II InP/CdS QDs (Dennis et al., 2012a) that have been shown by us and others to exhibit shell-thickness-dependent suppressed-blinking behavior in the NIR (Dennis et al., 2012a; Smith et al., 2017).

In this work, we address the role of core size, shell composition, and shell thickness on tuning the PL emission colors and radiative lifetimes of type-I and type-II systems with an InP core. A range of visible and NIR emitters have been produced by synthesizing core/shell QDs using three different sizes of core InP nanocrystals (small, medium, and large), four different shell compositions (ZnS, ZnSe, CdS, and CdSe), and varying the number of shell deposition iterations (1 through 10). A schematic of possible band alignments for each type of heterostructure is shown in Figure 1. In addition, we show the relative bulk conduction and valance band positions for each of the semiconductors used in this study. We demonstrate that in our cadmium-free type-I systems, the emission color is tuned throughout the visible wavelength range through variation of the core size alone, where the shell material acts as an optically inactive passivating layer, protecting the core NC. In our charge-separated type-II systems, the core size is less influential, while the shell thickness strongly impacts the emission peak wavelength and radiative lifetimes of the resulting NIR emitters.

## Materials and methods

### Materials

Oleylamine (OAm, 80–90%), 1-octadecene (ODE, 90%), diphenylphosphine (DPP, 98%), sulfur (S, 99.999%), tris(trimethylsilyl) phosphine (TMS_3_P, 98%), cadmium oxide (CdO, 99.95%) and hexanes (99%) were purchased from Fisher Scientific. Indium (III) acetate [In(Ac)_3_, 99.99%], oleic acid (OA, 90%), n-trioctylphosphine (TOP, 97%), selenium pellets (Se, 99.99%), zinc acetate [Zn(Ac)_2_, 99.0%], dioctylamine (98%), and ethanol (99.8%) were purchased from Sigma-Aldrich. ODE was heated to 120°C under vacuum for several hours prior to use and stored in an argon glovebox. All other chemicals were used directly without further purification. Quartz glass cuvettes were purchased from Starna Cells Inc. All air-sensitive materials were stored and handled in a glovebox under argon.

### Precursors

The metal and chalcogenide precursors were prepared with minor modifications to previously reported protocols. A 0.2 M stock solution of indium oleate with a molar ratio of 1:3 was prepared by mixing 3.2 mmol indium (III) acetate (0.93 g) and 10.61 mmol (3.35 mL) oleic acid and heating the reaction flask to 120°C for 2 h with multiple argon backfill and evacuation cycles to ensure complete removal of acetic acid. After a clear and colorless solution was obtained, 12.58 mL degassed ODE was added to the solution. Once the temperature of the solution reached 120°C, it was cooled to 90°C and vacuumed for another hour. A 0.2 M stock solution of cadmium oleate (Cd:OA 1:4) was prepared by mixing 7 mmol CdO (0.9 g) and 31.15 mmol (9.83 mL) oleic acid. The rust colored solution was degassed at 110°C for 1 h, backfilled with argon, and heated to 280°C until a clear and colorless solution was obtained. This solution was cooled to 100°C and vacuumed for an hour to remove any water produced during the cadmium oleate formation reaction. The flask was backfilled with argon and stored at 80°C for future use. A 0.2 M stock solution of zinc oleate (Zn:OA 1:4) was prepared by mixing 20 mmol zinc acetate (4.39 g) with 88.88 mmol (28.05 mL) oleic acid and degassing under vacuum at room temperature until no vigorous bubbling was observed. The temperature was slowly raised to 120°C and maintained for 2 h, until the solution was clear and no bubbling was observed. At this point, 71.95 mL degassed ODE was added to the solution. The flask was stored at 60°C for further use. Solutions of sulfur in both TOP and ODE were used as sulfur precursors. For the preparation of a 0.2 M stock solution of sulfur in ODE, 3.11 mmol (0.1 g) elemental sulfur and 15.59 mL ODE was mixed and heated to 80°C under vacuum for 2 h. The flask was backfilled with argon and heated at 80°C overnight to ensure complete dissolution of sulfur in ODE, and stored at the same temperature for future use. A similar procedure was followed for the preparation of the 0.2 M stock solutions of sulfur in TOP and selenium in TOP. TOP:S and TOP:Se were stored at room temperature in an argon glovebox for future use.

Diphenylphosphine selenide (DPP:Se) was synthesized as previously described (Evans et al., 2010). In an argon-filled glovebox, 20 mmol (3.48 mL) DPP, 20 mmol (1.58 g) selenium, and 25 mL anhydrous toluene were loaded in a 3-necked round bottom flask attached to a Findenser (Radleys, UK). The flask was heated at 110°C for 16 h. Following the removal of toluene in a rotary evaporator, white crystalline precipitates of DPP:Se were obtained. The collected precipitate was washed with cooled toluene to remove impurities. A 0.2 M stock solution of DPP:Se was prepared by mixing 8 mmol (2.12 g) of this powder and 39.98 mL ODE and heating to 100°C until the crystals completely dissolved in ODE. The solution was kept at 80°C to maintain solubility.

### Synthesis of InP core nanocrystals

InP core nanocrystals of three different sizes–small, medium, and large (S, M, and L, respectively) were prepared using hot injection synthesis. For the synthesis of the small and medium cores, 2 mL 0.2 M In(OA)_3_, 1.5 mL OAm, and 2 mL ODE were loaded into a 100 mL round bottom flask. The indium oleate solutions were heated under vacuum to 120°C and backfilled with argon multiple times, while syringes of 0.1 M and 1 M (TMS)_3_P in ODE were prepared in the glovebox. An initial 0.2 mL of 1 M (TMS)_3_P was rapidly injected into each flask of indium oleate at 191°C with the thermocouple temperature set to 178°C for the growth phase of the reaction. Starting within 45 s of the first injection, 2 mL of 0.1 M (TMS)_3_P was added to the flask via dropwise injection. Small cores were reliably produced by quenching this reaction after 3.5 min by removing the heating mantle and adding 12 mL of degassed, room temperature ODE. To generate medium cores, more indium and phosphorus was repeatedly added to the InP cores to provide precursors for growth with a total of 6 mL of warm 0.2 M In(OA)_3_ and 12 mL of 0.1 M (TMS)_3_P added dropwise in 4 separate aliquots each over the span of 20 min. After 32.5 min of total reaction time, the core growth was quenched by removing the heating mantle and adding 24 mL of degassed, room temperature ODE. The large InP cores were produced similarly to the small core protocol described above, except that the OAm was replaced by 0.5 mL dioctylamine and the reaction proceeded at 178°C for 14 h.

For the synthesis of InP/ZnS and InP/ZnSe, core InP QDs were used in their original reaction solution. For the synthesis of InP/CdS and InP/CdSe, core nanocrystals were cleaned by a precipitation and resuspension procedure. To clean the reaction product, aliquots were diluted in hexane and centrifuged to remove any precipitates in an argon glovebox. In a clean centrifuge tube, ethanol was added to the supernatant to precipitate the QDs, and the sample was centrifuged again. At this step, the clear supernatant was discarded and the colored precipitate was resuspended in hexane. A final centrifugation step removed any residual precipitates. The transparent, but colored, supernatant was used for the synthesis of InP/CdS and InP/CdSe. The same procedure was performed for cleaning the core/shell reaction products for further characterization.

### Synthesis of core/shell nanocrystals

Four distinct shell compositions comprising CdSe, CdS, ZnSe, and ZnS were deposited on the InP core nanocrystals using a modified SILAR technique, adapted for colloidal synthesis (Li et al., 2003). In this approach, aliquots of cationic and anionic precursors were added to the core solution between long anneals at growth temperatures (150–240°C). The required amounts of anion and cation precursor were calculated based on the number of surface atoms needed to add a monolayer to a certain size of a core/shell nanocrystal. For each reaction, 1 mL OAm and enough ODE to make up a total reaction volume of 10 mL were mixed in a 100 mL round bottom flask on a Schlenk line. The flask was vacuumed at room temperature for 30 min before the solution temperature was raised to 120°C for multiple vacuum/argon cycles. To protect the InP cores from Ostwald ripening caused by sudden exposure to heat, the flask was cooled to 60°C for the addition of 200 nmol of InP cores.

The flasks were kept under dynamic vacuum for an additional 15–20 min before the reaction temperature was raised. In addition, the reaction flask for InP/ZnSe was spiked with 0.21 mL (1.207 mmol) of DPP at this time. For all shell compositions, the cation precursor for the first deposition was injected at 150°C; the first anion injection proceeded 10 min later at the same temperature. Immediately after the anion injection, the temperature was raised to 240°C and the solution was annealed as described in Table 1. For InP/CdS and InP/CdSe heterostructures, all subsequent shell precursors were injected dropwise at 240°C (Dennis et al., 2012a). However, for InP/ZnS and InP/ZnSe heterostructures, a temperature cycling procedure was followed as previously described (Xie and Peng, 2009). Specifically, after the anion annealing at 240°C, the temperature of the flask was lowered to 150°C for the subsequent cation and anion injections before the temperature was again increased to 240°C for a longer anneal. To monitor the progress of the reaction, aliquots of the reaction solutions were taken at regular intervals. The reaction was terminated by cooling the solution to room temperature.

**Table 1:** Summary of shell reaction parameters.

	**Shell composition**			

	**ZnS**	**ZnSe**	**CdS**	**CdSe**
Core Preparation [solvent]	Raw [ODE]	Raw [ODE]	PR [hexanes]	PR [hexanes]
Cation Precursor [mol/L]	Zn(OA)_2_ [0.2]	Zn(OA)_2_ [0.2]	Cd(OA)_2_ [0.2]	Cd(OA)_2_ [0.2]
Anion Precursor [mol/L]	TOP:S [0.2]	DPP:Se [0.2]	S/ODE [0.2]	TOP:Se [0.2]
Cation/Anion Anneal Time [hr]	0.25/1.5	0.25/1.5	1/2.5	1/2.5
Injection/Annealing Temp. [°C]	150/240	150/240	240/240	240/240

### QD characterization

Absorption spectra were obtained using a NanoDrop 2000c spectrophotometer, using the pedestal. Samples were cleaned by precipitation and resuspended in hexane or TCE (to eliminate hexane reabsorption in NIR measurements) for PL measurements and measured in quartz cuvettes. Photoluminescence spectroscopy below 900 nm was measured with a Horiba NanoLog spectrofluorometer (Horiba Jobin Yvon) outfitted with a 450 W Xenon arc lamp, double excitation and double emission monochromators, and silicon CCD detector. NIR emission peaks were measured with a similar instrument outfitted with a liquid nitrogen-cooled InGaAs CCD array. Spectra were taken with 400 nm excitation and a 450 nm, 550 nm, or 800 nm longpass emission filter, depending on the wavelength region being examined. The collected spectra were corrected for lamp power and detector sensitivity using instrument-specific correction files.

Absolute quantum yield values were obtained using the spectrofluorometer described above in conjunction with a Quanta-phi six-inch integrating sphere (HORIBA Jobin Yvon). Cuvettes were loaded with hexanes in order to generate a blank reference following excitation at 400 nm. QD samples were diluted directly into the blanked cuvettes and measured with identical conditions. The absolute quantum yield (i.e., the photons emitted divided by the photons absorbed) was determined using the Horiba software.

Transmission electron microscopy (TEM) images were acquired using a JEOL 2100 LaB6 high-resolution microscope operating at 200 kV. Each sample was precipitated and resuspended in hexane twice and drop-cast on a copper TEM grid, which was washed successively by hexane, ethanol, and DI water. The grid was then gently heated to evaporate any residual water. TEM images were sized manually using ImageJ. TEM images have been size matched for presentation and the reported sizing information is based on 108–346 QDs, depending on the sample. The shell thickness was calculated by taking the difference between the core/shell radius determined via TEM and the core radius determined using the 1S absorption peak position (Xie et al., 2009).

Photoluminescence lifetime measurements were performed using an Edinburgh Instruments DeltaFlex time-correlated single photon counting (TCSPC) fluorescence lifetime instrument. QD samples were suspended in hexanes and loaded into quartz cuvettes. Samples were excited with a 405 nm EPL picosecond pulsed diode laser using a 1 microsecond pulse period. For each sample, the measurement continued until 10,000 photons were collected in the highest-intensity bin to ensure accurate fits. Photons were collected at the emission peak with a bandwidth window of 15–50 nm, depending on the FWHM of the sample. After collection, data was analyzed with F980 software (Edinburgh Instruments) by fitting the tail to the tri-exponential equation $R\left(t\right)=A_{1}e^{-t/\tau_{1}}+A_{2}e^{-t/\tau_{2}}+A_{3}e^{-t/\tau_{3}}$, where *A*_*n*_ is the percent weight of each exponential. The average photoluminescence lifetime, $\bar{\tau }$, was calculated using the following equation (Lakowicz, 2006; Jones and Scholes, 2010):

$$\begin{array}{l} \bar{\tau }=\sum_{n}\frac{A_{n}\tau_{n}^{2}}{\underset{m}{\sum }A_{m}\tau_{m}} \end{array}$$

### Effective mass model of core/shell heterostructures

The electronic structures of the heterostructures were calculated using a two-band effective mass model applied to each core/shell composition as has been previously described (Efros et al., 2000; Piryatinski et al., 2007; Dennis et al., 2012a). The confinement potential has been parametrized using values for the bulk bandgaps, conduction band energies, electron and hole effective masses, and material dielectric constants listed in Table S1. The 1S exciton energy is evaluated as a sum of the electron and hole 1S state energies due to the confinement potential (kinetic energies) plus their Coulomb binding energy (perturbative corrections).

## Results and discussion

**Figure 2:**
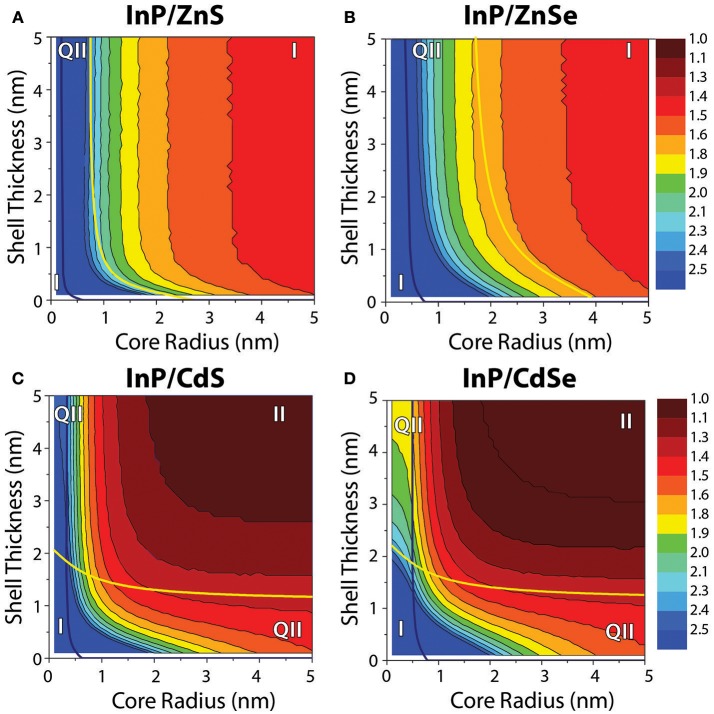
Contour plots depicting the bandgap energies of InP/ZnS, InP/ZnSe, InP/CdS, and InP/CdSe core/shell heterostructures as a function of the core radius and shell thickness, as predicted by effective mass modeling. In each case, the blue line denotes the boundary between regimes for which the hole is either delocalized over the entire heterostructure volume (left of the line) or core-localized (right of the line). For **(A)** InP/ZnS and **(B)** InP/ZnSe, the yellow line separates the regimes where the electron is delocalized over the entire particle volume (left of the line) from the regime where the electron is core localized (right of the line). For **(C)** InP/CdS and **(D)** InP/CdSe, the yellow line again indicates changes in the electron localization, separating the regimes where the electron is delocalized over the entire particle volume (below the line) from the regime where the electron is shell localized (above the line). The plots include notation indicating the regions of core/shell dimensions that correspond to different electron and hole localization regimes, indicating that the charge carriers are co-localized [type-I (I)], spatially separated [type-II (II)], or that one charge carrier is localized while the other is delocalized [quasi-type-II (q-II)]. The same color scale is used for all four plots.

In order to examine the impact of the composition and dimensions of InP-based core/shell heterostructures on their optical properties, we used an effective mass model to generate wavefunctions for the electron and hole carriers as a function of the core and shell compositions and dimensions and, from the overlap integral, predict the bandgap energy of the heterostructure (Figure 2). The modeling data predict that InP/ZnS heterostructures with core diameters larger than 2 nm are exclusively type-I emitters with both the electron and hole localized in the core. The vertical bands of equal energy show that the final emission color is primarily dictated by the starting core size and uninfluenced by ZnS shell thickness. The InP/ZnSe data similarly exhibit vertical bands in the bandgap energy plot after a shell ~1 nm thick is deposited, but a quasi-type-II structure is predicted for core sizes up to 4 nm in diameter—a range that is more relevant for this study than the very small core sizes predicted to exhibit quasi-type-II behavior for the InP/ZnS system. In this context, we would expect the core size to primarily dictate the color of both InP/ZnS and InP/ZnSe heterostructures. The InP/ZnSe particles, however, are predicted to exhibit more red-shifting with the deposition of thin shells than InP/ZnS. After deposition of several atomic monolayers, neither material is expected to show photophysical changes as a function of shell thickness.

In contrast, modeling of the InP/CdS(e) systems predicts a strong influence of both core size and shell thickness on the resulting bandgap. In these systems, larger cores naturally exhibit redder emission, but the bandgap energy also shifts quickly and significantly with CdS(e) shelling. The hole wavefunction is shown to localize in the core in all cases where the core is >1 nm in diameter. The electron wavefunction on the other hand indicates localization of the electron in the shell as soon as the shell is >1 nm (1.5 nm) for InP/CdS(e). Because of these clear localization patterns with such small core sizes and relatively thin shells, nearly all of the InP/CdS(e) parameter space is classified as falling into a type-II localization regime. One of the hallmarks of a type-II system is that it can exhibit radiative recombination at photon energies lower than is possible for either the core or shell material in isolation. Although not all of the bandgaps predicted for the InP/CdS(e) core/shell structures in the type-II regime are lower energy than the bulk bandgap of InP at 1.35 eV, these composition and dimension combinations are still type-II, as they exhibit lower energy bandgaps than would be possible for either InP or CdS(e) of the same size (i.e., with confinement).

It is well established that semiconductor nanocrystals smaller than their exciton Bohr radius are quantum confined and exhibit size-dependent optoelectronic properties (Murray et al., 1993; Battaglia and Peng, 2002). This was seen for the InP core particles generated for this study as well. InP cores were nucleated by injecting the highly reactive phosphorus precursor, tris(trimethyl silyl)phosphine, into an indium oleate solution heated to 191°C. Oleylamine facilitated the fast, uniform growth of the smallest cores; additional indium and phosphorus precursors were added at the growth temperature (178°C) to yield the medium cores. The large cores were synthesized overnight at 178°C in the presence of a secondary amine (di-n-octylamine) without further precursor addition. The resulting cores exhibit exciton 1S peaks (small and medium) or shoulders (large) at 445, 543, and 622 nm, which correlate to particle diameters of 1.5, 2.5, and 3.7 nm, respectively (Figure S1) (Xie et al., 2009).

**Figure 3:**
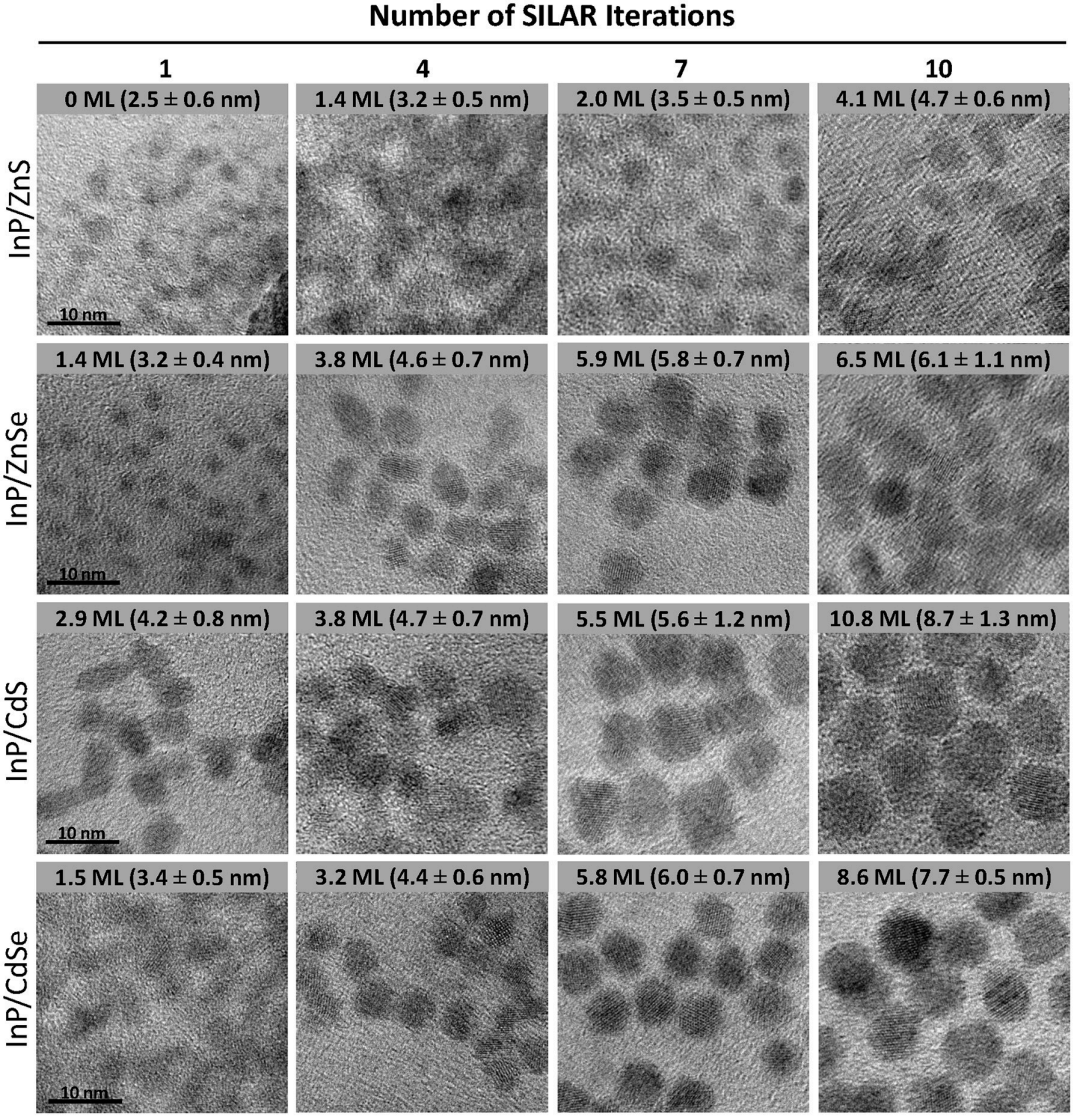
Size-matched TEM images of InP/ZnS, InP/ZnSe, InP/CdS, and InP/CdSe core-shell heterostructures after 1, 4, 7, and 10 successive shell depositions. The scale bar for all images is 10 nm. The average ± standard deviation diameter of the core/shell particles is included in the inset text in the corresponding image. The size statistics are based on *n* = 108–346 particles, depending on the sample.

To observe the effect of shell composition and thickness on the optical properties of InP, epitaxial shells of four different semiconductor materials (ZnS, ZnSe, CdS, and CdSe) were grown on each of three sizes of InP cores with shell growth confirmed through TEM imaging (Figure 3). Lattice fringes are evident in the images, demonstrating the crystallinity of the nanoparticles. The core sizes determined from the 1S peak position and the average particle diameters obtained from TEM sizing data (Figure S2; Table S2) were used to determine the average shell thickness for each sample, assuming spherical core and core/shell particles. The number of shell monolayers (MLs) deposited was determined by dividing the shell thickness in nm by the thickness of one cation/anion layer (i.e., half the lattice constant of the shell material; Table S1).

**Figure 4:**
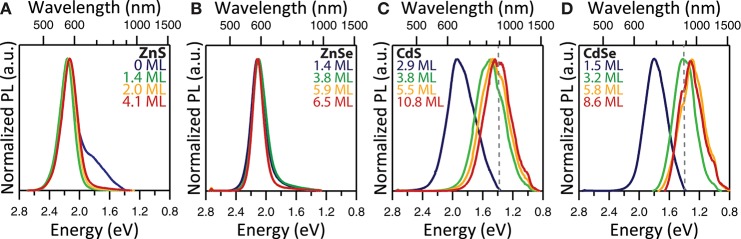
Normalized PL emission spectra of **(A)** InP/ZnS, **(B)** InP/ZnSe, **(C)** InP/CdS, and **(D)** InP/CdSe for medium sized cores after 1, 4, 7, and 10 SILAR iterations. The resulting thickness of the shell in atomic monolayers is indicated in the figure legends. The dotted gray line indicates a detector change at 897 nm.

It is apparent from an examination of the sizing data (Figure S2; Table S2) that the ZnS shell deposition proceeded significantly less efficiently than the deposition of the other shell materials. While the ZnS monolayer is the thinnest of the four materials with a lattice constant of 5.41 Å (Table S1), this difference in unit cell size does not account for all of the difference in the final size. The ZnS and ZnSe shells are only 4.1 and 6.5 MLs thick, respectively, after 10 iterative rounds of SILAR deposition. For the cadmium-free systems, the shell growth procedure is not as effective as that of the cadmium-containing systems for which the chemistry is very well developed. This is mainly attributed to the less reactive nature of the zinc precursor compared to the cadmium precursors. The impact of this lower reactivity can be observed in the PL spectra as well, particularly for some InP/1ZnS(e) samples (Figure 4; Figure S3), which exhibit low energy tails indicative of trap emission. This suggests incomplete formation of the ZnS(e) shell in the early stages of the reaction. This tail disappears and the full width at half maximum (FWHM) of the PL peak decreases with subsequent shelling (Figure S4), providing evidence for complete shell formation with successive precursor addition. We suspect that a combination of high lattice strain, due to the large lattice mismatch between InP and ZnS (7.8%), and the lower reactivity of the zinc precursor combines to produce the least efficient shell deposition in the case of ZnS. Several reports describe increasing the reactivity of zinc by means of the addition of phosphorous containing compounds, with the aim of the formation of more reactive zinc phosphine complexes, particularly for the synthesis of thick-shelled InP/ZnSe heterostructures (Joo et al., 2009; Evans et al., 2010; Yu et al., 2011).

To improve the efficiency of the ZnS(e) shell depositions, several Se and S precursors were tested for effective shell growth with zinc oleate. Both trioctylphosphine sulfide (TOP:S) and sulfur dissolved in the non-coordinating solvent octadecene (S/ODE) were tested for ZnS deposition. The addition of sulfur caused significant etching of the core nanocrystals at elevated temperatures, especially in the first stages of the synthesis, as seen through the blue-shifting of the 1S exciton peaks (Figure S5). Using TOP:S, however, the 1S peak remained stable, indicating that the core nanocrystal remained intact. The selenium precursors tested include trioctylphosphine selenide (TOP:Se) and diphenylphosphine selenide (DPP:Se). When DPP:Se was used, TEM images indicated QDs of a larger average diameter and a more uniform morphology compared to TOP:Se (Figure S6). This is likely due to the formation of the more reactive zinc phosphine monomers, resulting in a higher reaction yield and a more effective shell growth with increasing shell thickness (Yu et al., 2011). We found that spiking the reaction flask with 0.21 mL (1.207 mmol) of DPP in addition to using DPP:Se as the anionic precursor improved the overall quality of the InP/ZnSe QDs (Figure S6).

For CdS(e) shell formation, successful shell growth was observed via TEM imaging using S/ODE and TOP:Se as anionic precursors, respectively, with cadmium oleate as the cationic precursor. Little optimization of the SILAR protocol was needed for CdS(e) shell growth, as many of the thick-shell examples in the literature focus on cadmium systems (Zhou et al., 2016, 2017; Niu et al., 2017). The primary struggle in adapting these protocols to the InP-based system hinged on deposition of the first shell layer without excessive etching or dissolution of the InP core. This is successfully achieved by lowering the initial shell growth temperature to 150°C, as we previously reported (Dennis et al., 2012a).

**Figure 5:**
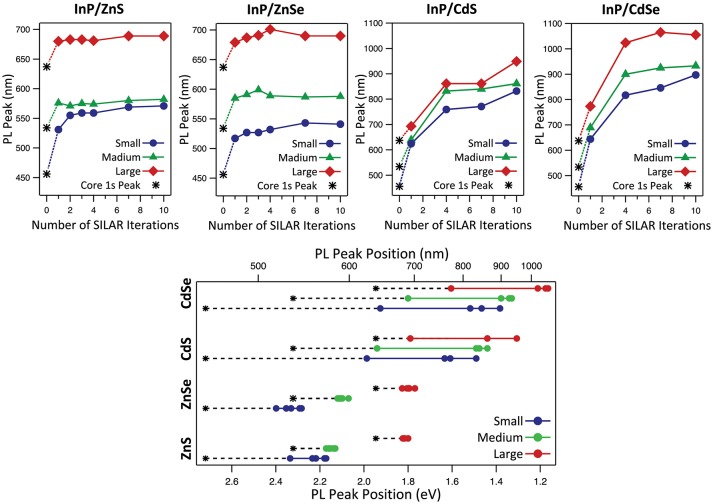
Variation of PL peak position with increasing shell thickness for InP/ZnS, InP/ZnSe, InP/CdS, and InP/CdSe heterostructures with small, medium, and large cores. The asterisk denotes the exciton 1s absorption peak of the InP cores.

Optical characterization was performed on samples comprising three core sizes and four shell thicknesses for each of the four core/shell heterostructure compositions. This set of forty-eight samples spans a wide range of particle sizes, emission wavelengths, and photoluminescent lifetimes, demonstrating the interplay between the core/shell structure and the photophysical function. By examining the PL peak position, we immediately observe the impact of the different band alignments (Figures 4, 5, Figure S3). InP/ZnS and InP/ZnSe heterostructures have shell materials with significantly larger bandgaps than their core material (Table S1) and thus exhibit type-I behavior. Both electrons and holes are confined to the core nanocrystal, providing the exciton with protection against oxidation and photobleaching. We can see that the PL peak positions of type-I heterostructures do not red-shift in spite of successful shelling demonstrated by an increase in the average diameter of the samples. The thicker shell deposition is, however, accompanied by a slight decrease in the FWHM of these samples, which demonstrates improved passivation of the core and the absence of electron leakage into the shell. The trap emission evident as a red tail present in some PL spectra indicates incomplete shelling after the deposition of the first ZnS(e) monolayer, but disappears following subsequent shell additions.

Although the modeling results predict stronger confinement for the InP/ZnS system compared to InP/ZnSe, we did not find this to be evident in the PL spectra. During initial experiments, InP/ZnS synthesized using S/ODE exhibited measurably bluer emission than InP/ZnSe synthesized with TOP:Se, but visually observable color shifts during the addition of the sulfur was indicative of core etching (Figure S5). Once the sulfur precursor was changed to TOP:S to mitigate etching as discussed above, the difference in the peak PL emission wavelength between the InP/ZnS and InP/ZnSe generated with the same starting cores narrowed. Our modeling predicts that smaller cores will exhibit a more quasi-type-II behavior in the InP/ZnSe system than larger cores, which are predicted to be type-I (Figure 2). We would expect this to present as a larger red-shift for InP/ZnSe with smaller core sizes, which we observe in fact for both InP/ZnS and InP/ZnSe systems (Figure 5). The red-shift for the small-core InP/ZnS was significantly larger than expected compared to other reactions. This reaction may have been affected by Ostwald ripening, which is more likely with smaller cores than larger ones, prior to successful deposition of the ZnS shell.

Based on the position of the exciton 1S peak of the cores and taking into consideration the average Stokes shift of 60 nm associated with InP, both InP/ZnS and InP/ZnSe largely exhibit type-I behavior (Mićić et al., 1996; Fu and Zunger, 1997). Both the EMA model and our empirical data show that for the type-I systems, the starting core size is the determining parameter in tuning the emission color. The vertical contour plot lines for InP/ZnS(e) in Figure 2 indicate that quantum confinement and the heterostructure bandgap do not change with additional ZnS(e) shell thickness after the initial deposition of a limited shell. Our graphs of InP/ZnS(e) peak position with respect to SILAR iterations in Figure 5 confirm this behavior empirically as well. Our results confirm what others have observed—namely that InP/ZnS(e) emission wavelengths are tunable throughout the visible wavelength range by altering core size.

In contrast with the type-I heterostructures, deposition of CdS and CdSe on the InP core is accompanied by a significant red-shifting of the PL peak into the NIR wavelength ranges, which plateaus after the 7th round of SILAR deposition. Using CdSe or CdS as the shell material, the PL was successfully tuned throughout the red and into the near-infrared wavelength ranges. Larger core sizes resulted in emission at even longer wavelengths. In type-II systems, the significant influence of shell thickness on emission tuning is due to the separation of charge carriers: the confinement of holes in the core and electrons in the shell. In both cadmium shell systems, carrier recombination can take place across the core/shell interface at a lower energy than the bandgap of either of the constituent semiconductor materials.

It is important to note that in addition to the expected type-II NIR emission from the InP/CdS(e) structures, thicker shelled (>3 ML) InP/CdSe—but not InP/CdS—also exhibited shell-thickness dependent *visible* emission (Figure S7). In an extensive study that is beyond the scope of this paper (Buck et al., in preparation), we demonstrate that both of these photon energies (visible and NIR) are emitted by a single particle, appearing to originate from the type-II recombination described here, as well as, recombination events where both electron and hole carriers are confined in the CdSe shell. It is further notable that this behavior has not been observed in the InP/CdS system. It is likely that the core etching observed upon addition of S/ODE during the InP/ZnS synthesis also occurs when S/ODE is added for CdS deposition. This remodeling of the InP surface could impact surface states, preventing the visible emission originating in the shell. While we have spectroscopic evidence of the InP/ZnS etching (Figure S5, discussed above), we can only surmise that the S/ODE behaves similarly during the InP/CdS synthesis because the red-shifting of the optical features is pronounced even at the earliest depositions.

**Figure 6:**
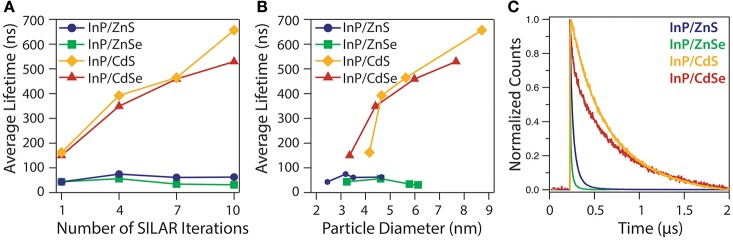
PL lifetime measurements of InP/ZnS, InP/ZnSe, InP/CdS, and InP/CdSe heterostructures synthesized using medium sized cores: average excited state lifetime relative to **(A)** the number of SILAR iterations and **(B)** the particle diameter, as determined by TEM. Lines drawn as a guide to the eye. **(C)** PL decay curves of the largest core/shell heterostructures from each composition.

The contrast between the type-I and type-II systems is pronounced in the radiative lifetimes of the heterostructures, as well as, in the PL energy. Figure 6 illustrates the PL lifetime for the thickest shell of each heterostructure, as well as, the change in lifetime with shell thickness. The lifetime measurements were taken for the emission range comprising one third of the total emission peak width in order to focus on bandgap emission rather than the redder trap state emission visible in the thin-shelled InP/ZnS(e) samples. The InP/ZnS(e) heterostructures exhibit negligible change in PL lifetime with additional shell deposition, as the carriers are both confined in the InP cores; the radiative recombination occurs relatively quickly and the average lifetime does not exceed 100 ns. The InP/CdS(e) structures exhibit dramatically longer lifetimes than the InP/ZnS(e) structures, consistent with type-II behavior. In addition, InP/CdS(e) QDs exhibit a shell thickness-dependent lengthening of the lifetime as the excited electron spreads through a larger shell volume with PL lifetimes approaching 700 ns. Even at particle diameters between 3 and 6 nm, where the particle size across different heterostructures are similar, the radiative lifetimes can be up to an order of magnitude higher for the InP/CdS(e) systems than for InP/ZnS(e).

To see shell-thickness dependent trends in PL intensity, we plotted the absorbance-normalized PL spectra of the medium core samples for four shell thicknesses of each of the four shell compositions (Figure S8). For InP/ZnS and InP/ZnSe, the brightest samples are obtained after the deposition of 1.96 and 3.81 shell MLs, respectively. We observe a clear increase in brightness as the InP surface is completely passivated, but thicker shells result in decreased PL intensity, presumably due to strain effects and defect formation. It's notable that this decline is observable at thinner ZnS shells than ZnSe shells (4.1 vs. 5.9 MLs, respectively); this difference correlates with the higher lattice mismatch for InP/ZnS vs. InP/ZnSe (7.8 vs. 4.9%). For the InP/CdS(e) samples, the first round of shell deposition passivates the InP core, enabling relatively bright emission from the previously non-emissive core. With subsequent shell deposition, however, the effects of spatial charge separation are observed as a significant decrease in the PL intensity. This decrease in PL intensity trends with the increase in PL lifetime as a result of the type-II nature of the heterostructures.

While the trends within each shell composition group are very consistent, we were also interested in the differences in brightness amongst compositions. Here, it is important to note that the absorbance-normalized PL measurements were taken many months after the particles were synthesized. We qualitatively observed that the InP/ZnSe samples significantly dimmed with time through casually monitoring brightness by eye under UV illumination, an option that is not feasible for the NIR-emitting InP/CdSe samples. The thinner-shelled InP/ZnS samples also lost some of their PL intensity with age: months earlier, both the 1.4 and 2.0 ML InP/ZnS exhibited absolute QYs of 43–45%, whereas at the time of the absorbance-normalized PL measurements, the 2.0 ML sample still exhibited a 43% QY, but the relative emission of the 1.4 ML sample had decreased (Figure S8). It is challenging to compare the InP/ZnS(e) samples to the InP/CdS(e) samples because of the different detector used for the NIR PL measurements—i.e., the y-axis scaling between the InP/ZnS(e) and InP/CdS(e) plots in Figure S8 is quite different. We'll note, however, that we were never able to take absolute QY measurements of even the thin-shelled InP/CdS(e) systems on our instrument because the particles were so dim. The high absorptivity of the InP/CdS(e) samples is helpful for brightness, but the QY measurements require very dilute/low absorbing samples, making it more challenging to get a measurement that we were confident in from the very dim samples. In this context, despite the different y-axes of the InP/ZnS(e) and InP/CdS(e) plots, we are quite confident in saying that the type-II heterostructures were considerably dimmer than the type-I QDs.

## Conclusions

We shine light on the optical tunability of InP-based core/shell heterostructures through compositional and dimensional modifications, resulting in a head-to-head comparison of type-I and type-II InP QDs. The emission energy of the InP/ZnS(e) type-I core/shell QDs was tuned by altering the core size, while the emission of the InP/CdS(e) type-II counterparts was primarily shell thickness-dependent. We demonstrate that charge separation in type-II systems caused by the valence and conduction band alignments gives rise to strongly shell thickness-dependent increase in average PL lifetimes compared to either thin-shelled InP/CdS(e) or InP/ZnS(e) of any shell thickness. Our systematic study of these InP core/shell heterostructures provides insight into QD design for a variety of biological and optoelectronic applications.

## Author contributions

AD and JH conceived of and designed the project. RT performed the experimental work and data analysis with assistance from AM for sizing of the TEM images. RI performed PL spectroscopy measurements in the NIR wavelength range. AP performed the effective mass modeling of the heterostructures. RT and AD wrote the manuscript.

### Conflict of interest statement

The authors declare that the research was conducted in the absence of any commercial or financial relationships that could be construed as a potential conflict of interest.
