# Supplementary material for: Bandgap Engineering of Indium Phosphide-Based Core/Shell Heterostructures Through Shell Composition and Thickness
Source: Front Chem. 2018 Nov 20;6:567. doi: 10.3389/fchem.2018.00567 (PMC6255924; doi:10.3389/fchem.2018.00567)
Supplement: Supplementary file 1 [file Data_Sheet_1.pdf]

Supplementary Material

Bandgap Engineering of Indium Phosphide-Based Core/Shell Heterostructures through Shell Composition and Thickness

Reyhaneh Toufanian[1], Andrei Piryatinski[2], Andrew H. Mahler[3], Radhika Iyer[4], Jennifer A. Hollingsworth[4], Allison M. Dennis[1,3,*]

[1] Division of Materials Science and Engineering, Boston University, Boston, MA, USA
[2] Theoretical Division, Los Alamos National Laboratory, Los Alamos, NM, United States
[3] Department of Biomedical Engineering, Boston University, Boston, MA, USA
[4] Center for Integrated Nanotechnologies, Materials Physics and Applications Division, Los Alamos National Laboratory, Los Alamos, NM, USA

[*] **Correspondence:** Allison Dennis**;** aldennis@bu.edu

Table S1. Properties of bulk semiconductor materials[1-3]

	InP	ZnS	ZnSe	CdS	CdSe
Crystal Structure	Zinc blende	Zinc blende	Zinc blende	Zinc blende	Zinc blende
Valence Band[a] [eV]	1.1	0	0.53	0.18	0.60
Conduction Band[a] [eV]	2.45	3.61	3.22	2.67	2.34
Bulk Bandgap [eV]	1.35	3.61	2.69	2.49	1.74
Electron Effective Mass[b] [m_e^*]	0.073	0.34	0.15	0.21	0.13
Hole Effective Mass[b] [m_h^*]	0.64	1.75	0.53	0.68	0.45
High Frequency Dielectric Constant	9.61	5.1	5.6	5.3	5.8
Lattice Constant [Å]	5.869	5.41	5.668	5.82	6.08
Lattice Mismatch[c] [%]	0	7.82	4.9	0.83	3.59
Bond Strength [kJ/mol]	197.9	205	136	196	310

[a] Energy in eV relative to vacuum.
[b] In units of m_e, i.e., 9.11×10^{-31} kg.
[c] Relative to the InP crystal lattice.

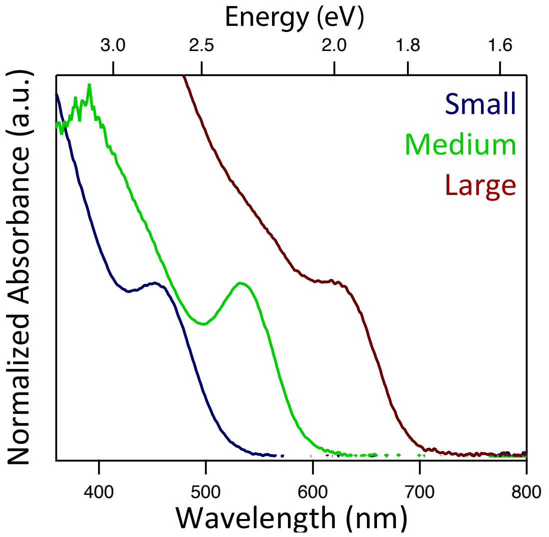

Figure S1. Absorbance spectra of small, medium, and large InP cores.

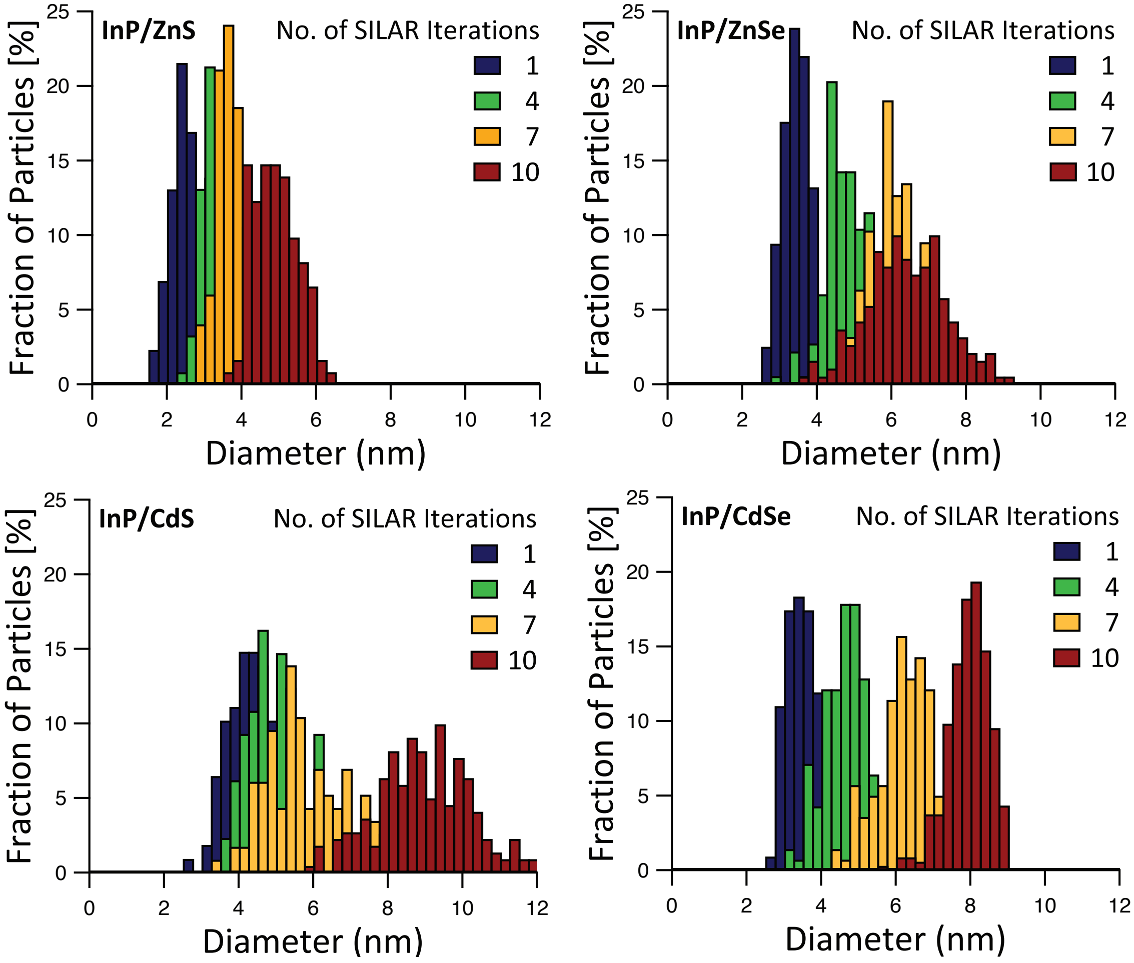

Figure S2. Histograms demonstrating size distribution of QD samples on the medium core after 1, 4, 7, and 10 SILAR iterations obtained through analysis of sizing based on TEM images.

Table S2. Sizing of core/shell particles with the medium core (2.45 nm diameter).

Composition	# of SILAR iterations	Diameter [nm]	+/- [nm]	+/- [%]	Shell Thickness [nm]	No. of Shell MLs	No. of Dots Sampled
InP/ZnS	1 ML	2.5	0.6	23.3	0	0.0	130
	4 ML	3.2	0.5	14.4	0.7	1.4	122
	7 ML	3.5	0.5	12.8	1.1	2.0	199
	10 ML	4.7	0.6	12.3	2.2	4.1	122
InP/ZnSe	1 ML	3.2	0.4	13.3	0.8	1.4	159
	4 ML	4.6	0.7	14.3	2.2	3.8	182
	7 ML	5.8	0.7	12.8	3.3	5.9	126
	10 ML	6.1	1.1	17.5	3.7	6.5	190
InP/CdS	1 ML	4.2	0.8	19.2	1.7	2.9	108
	4 ML	4.7	0.7	14.8	2.2	3.8	129
	7 ML	5.6	1.2	20.6	3.2	5.5	112
	10 ML	8.7	1.3	15.0	6.3	10.8	221
InP/CdSe	1 ML	3.4	0.5	15.8	0.9	1.5	109
	4 ML	4.4	0.6	13.4	2.0	3.2	140
	7 ML	6.0	0.7	12.4	3.5	5.8	140
	10 ML	7.7	0.5	7.0	5.2	8.6	346

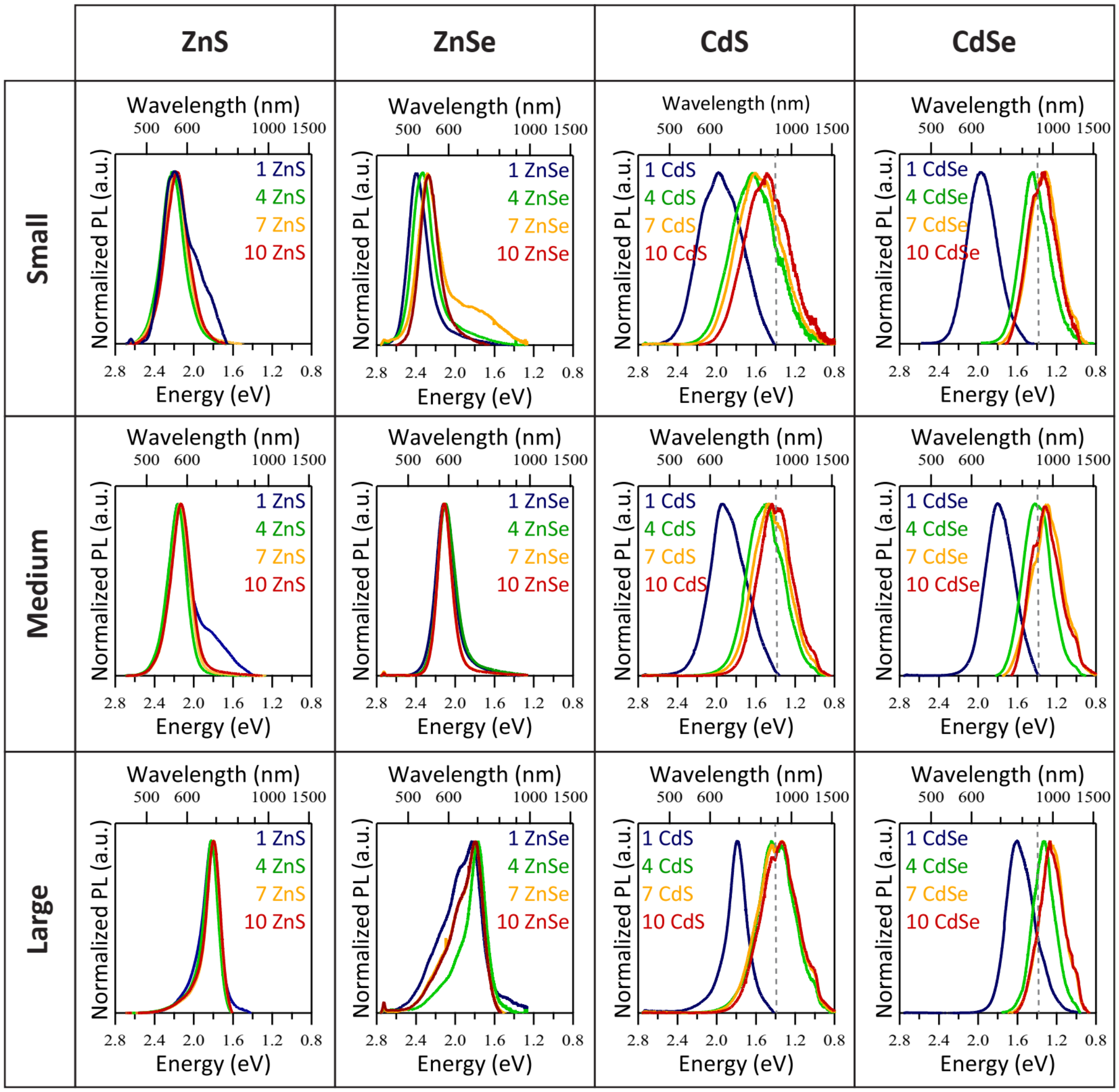

Figure S3. PL emission spectra of QDs synthesized using small, medium, and large (top to bottom) core InP nanocrystals after 1, 4, 7, or 10 SILAR iterations of ZnS, ZnSe, CdS, and CdSe (left to right). The dashed gray line indicates a detector change at 897 nm.

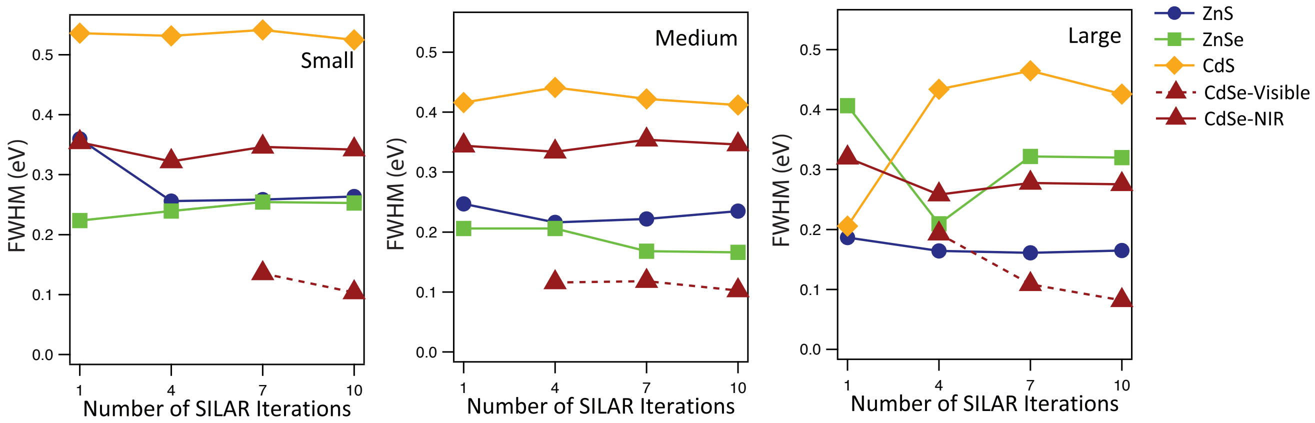

Figure S4. Full width at half maximum of the PL spectra of heterostructures synthesized using medium sized InP core nanocrystals. For the dual emitting InP/CdSe, the solid line depicts the NIR emission peak and the dashed line depicts the visible emission peak. For samples with medium and large cores, the visible peak is observed after four SILAR iterations, and for the small sample after seven SILAR iterations.

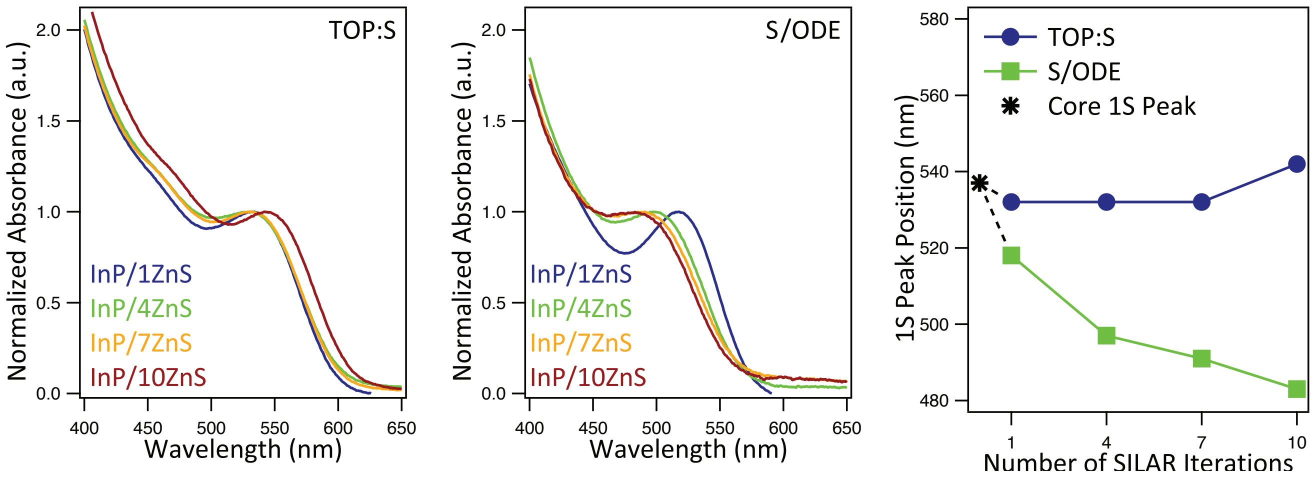

Figure S5. Comparison of the absorbance spectra and 1S peak positions of InP/ZnS samples synthesized using TOP:S and S/ODE after 1, 4, 7, or 10 SILAR iterations.

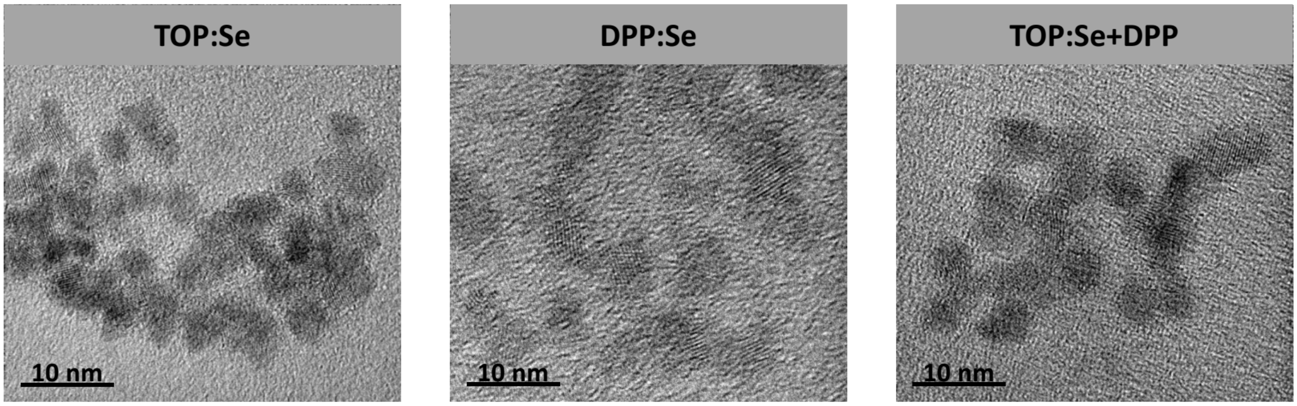

Figure S6. Comparison of the TEM images of InP/ZnSe samples synthesized using TOP:Se, DPP:Se, and TOP:Se in addition with DPP to study the effect of zinc-phosphine complexes on the formation of the ZnSe shell.

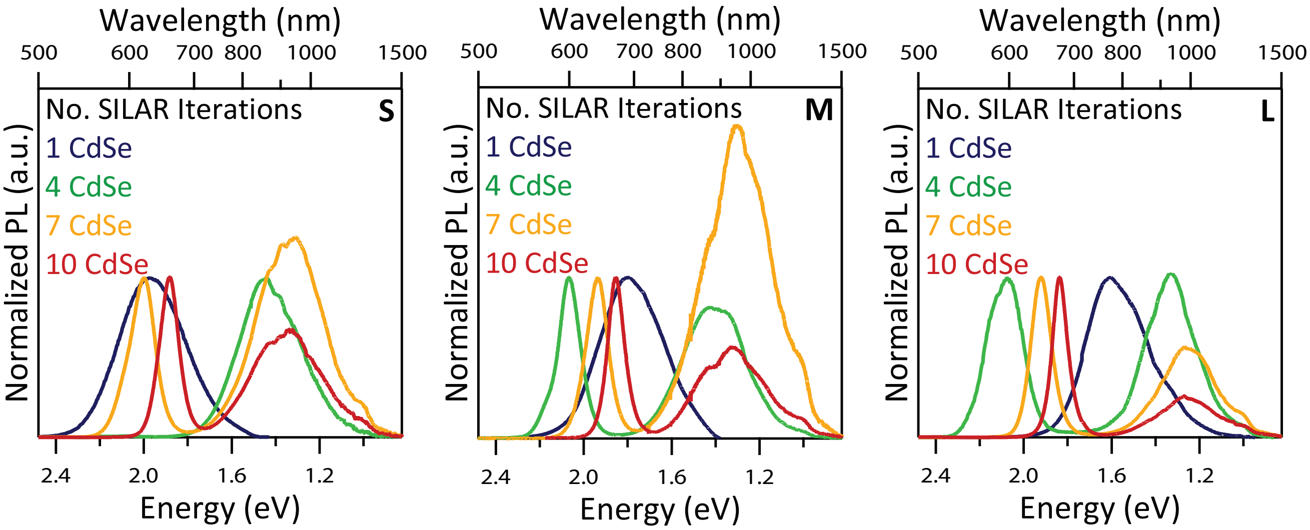

Figure S7. PL spectra of dual emitting InP/CdSe heterostructures synthesized using small, medium, and large cores (left to right) depicting a narrow emission peak in the visible and a broad emission peak in the NIR. The visible emission peaks have been normalized to one.

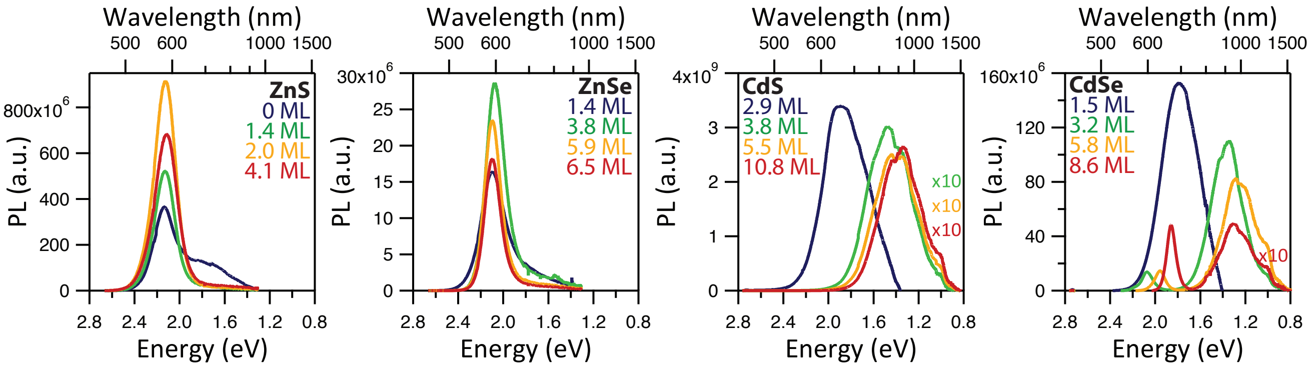

Figure S8. Absorbance-normalized PL spectra depict trends in relative brightness of the heterostructures with respect to shell thickness. Samples shown were synthesized using the medium-sized InP cores and 1, 4, 7, and 10 SILAR iterations. The legends indicate the number of shell monolayers deposited. PL spectra normalized to their absorbance at the excitation wavelength, 400 nm.

References:

1. Gardner, George S. "Handbook of Chemistry." *Journal of the Franklin Institute*, vol. 229, no. 1, 1940, doi:10.1016/S0016-0032(40)90947-4.
2. Reiss, Peter, et al. "Core/shell Semiconductor Nanocrystals." *Small*, vol. 5, no. 2, 2009, pp. 154–68, doi:10.1002/smll.200800841.
3. Wei, Su Huai, and Alex Zunger. "Calculated Natural Band Offsets of All II-VI and III-V Semiconductors: Chemical Trends and the Role of Cation D Orbitals." *Applied Physics Letters*, vol. 72, no. 16, 1998, pp. 2011–13, doi:10.1063/1.121249.
